# Supplementary material for: “Ad hoc policy decisions” in the news: Media framing analysis of a pesticide import ban in Sri Lanka
Source: PLOS Glob Public Health. 2024 Jul 16;4(7):e0003497. doi: 10.1371/journal.pgph.0003497 (PMC11251609; doi:10.1371/journal.pgph.0003497)
Supplement: S1 Table — (DOCX) [file pgph.0003497.s001.docx]

# S1 Table. Frame Sub-elements

| **Frame** | **Position** | **Cause** | **Consequence** | **Solution** |
| --- | --- | --- | --- | --- |
| Environmental impact | Supportive | Availability of pesticides  Use/overuse of pesticides | Excessive use of chemicals Negative environmental and/or health impact  Pollution of waterways  Soil infertility | Limit access to synthetic pesticides/fertilisers  Move to organic farming  Higher quality pesticides/fertilisers  Educate farmers  Phase out synthetic products progressively |
|  | Opposing | Farmers lack of knowledge  Vendors promote sales to increase profit | Excessive use of chemicals | Private-public partnerships  Limit access to synthetic pesticides/fertilisers |
| Farmer livelihoods | Supportive | Shortage of pesticides and fertilisers; | Farmers’ disappointment; | Locally produced products; |
|  | Opposing | Late cultivation season start  Shortage of pesticides and fertilisers | Crops destroyed  Farmers’ disappointment  Farmers and families struggling  High prices  Increased cost of production  Reduced earnings | New technologies and monitoring  Private-public partnerships  Revert the ban Make pesticides available to farmers  Phase out synthetic products progressively |
|  | Neutral | Farmers lack of knowledge  Shortage of pesticides and fertilisers | High prices  Abandoning or burning land | Make pesticides available to farmers |
| Food security | Opposing | Shortage of pesticides and fertilisers  Use/overuse of pesticides | Famine  Food shortages | Educate farmers  Make pesticides available to farmers  Move to organic farming  Phase out synthetic products over longer period |
| Human health | Supportive | Availability of pesticides  Use/overuse of pesticides  Vendors promote sales to increase profit | Negative health impact  NCDs  Negative environmental and/or health impact  Excessive use of chemicals | Move to organic farming  Phase out synthetic products over longer period  Stop import of chemical pesticides/fertilisers  Educate farmers |
|  | Opposing | Use/overuse of pesticides | Negative environmental and/or health impact | Responsible synthetic pesticide use |
| Industry livelihoods | Opposing | Shortage of pesticides and fertilisers | Chaos in agriculture sector  Crops destroyed  Famine  Lower quality of certain crops  Reduced crop yield  Reduction in export earnings  Yield loss  Food shortages | Make pesticides available to farmers  Phase out synthetic products over longer period |
|  | Neutral | Shortage of pesticides and fertilisers | Lower quality of certain crops  Reduced crop yield  Yield loss | Allow import for some crops (e.g. tea)  Make pesticides available to farmers |
| Organic farming | Supportive | Use/overuse of pesticides | High prices  Negative environmental and/or health impact  NCDs  Soil infertility | Move to organic farming  Educate farmers  New technologies and monitoring  Phase out synthetic products over longer period  Promote organic chemicals  Stop import of chemical pesticides/fertilisers  Timely planting of crops  Encourage natural formulations |
|  | Opposing | Shortage of pesticides and fertilisers  Yield loss | Yield loss  Famine | Phase out synthetic products over longer period  Higher quality agrochemicals |
| Pesticide market | Supportive | Shortage of pesticides and fertilisers | Smuggling and black market | Raids in markets |
|  | Opposing | Use/overuse of pesticides  Shortage of pesticides and fertilisers | High prices  No discount prices for pesticides  Smuggling and black market  Stockpiling to increase price | Make pesticides available to farmers; |
| Political decisions | Supportive | Sri Lanka’s financial problems | Famine |  |
|  | Opposing | Sri Lanka’s financial problems | Crops destroyed  Food shortages | Revert the ban |
