# Supplementary material for: “Ad hoc policy decisions” in the news: Media framing analysis of a pesticide import ban in Sri Lanka
Source: PLOS Glob Public Health. 2024 Jul 16;4(7):e0003497. doi: 10.1371/journal.pgph.0003497 (PMC11251609; doi:10.1371/journal.pgph.0003497)
Supplement: Attachment — Submitted filename: Responses to reviewers_R1.docx [file pgph.0003497.s002.docx]

| **Reviewer 1** | | |
| --- | --- | --- |
| **Reviewer comment** | **Response** | **Change in paper** |
| I find the article informative and generally well executed and well-written. This is a good example that value can be extracted in studies without having to resort to complex statistical analysis, provided researchers are still willing to do the necessary work. The approach taken, and the development of the coding framework, all seem well executed, and the limitation of not assessing the inter-rater reliability of the coding (primarily due to not all the coding being done in duplicate) is already acknowledged, but while this is a weakness, it does not render the results implausible. | We thank the reviewer for these positive and encouraging comments. |  |
| 1. Results (first paragraph). The table indicates 11 articles for policy end users, and not ten. | Thank you for identifying this, we have corrected this error. | Page 5 |
| 2. PLOS uses ICMJE referencing. According to ICMJE guidelines, foreign language sources should be referenced in the original language, with an (optional, but I think recommended) translation in brackets. See <https://www.nlm.nih.gov/bsd/uniform_requirements.html> This also might clarify the odd title to reference 35, which reads "...there will be famine and famine in the country..." which I assume should be "food shortage and famine". | We thank the reviewer for this and we have added original language titles for all Sinhala and Tamil articles and corrected the translation of the reference mentioned. | Reference list |
| There are also typos in references 2, 32, 37. | Thank you, we have double checked all translations before submitting the revised article. | Reference list |
| 3. There is a small language error in Figure 2, where I suspect it should read "the impact of the ban on the pesticide market..." instead of "the impact of the ban has on the pesticide market" | Thank you – we have corrected this error. | Figure 2 |
| 4. Environmental impact section: the last sentence needs a citation. | Thank you, we have added this citation to the manuscript. | p. 11 |
| 5. Pesticide market section: Delete the word 'how' from "One article described how raids in market to address smuggled products, yet this article did not present a broader long-term solution for the problems created by the imposed import ban." | We have deleted this. |  |
| 6. Political decisions section: Recommend changing 'one' to 'that' in the sentence "The author of one article, who was a former minister for agriculture, " | We have amended this, thank you. |  |
| The only larger recommendation I could make is that I think the authors missed a key point regarding the fact that many articles appear to have included multiple framings (They note: "We identified eight different framings of the import ban in the 102 included articles analysed for the study. These framings were used a total of 139 times, with each of the framings differing in the number of times they were identified."). I suspect the combination of framings itself would also have proved insightful, especially in light of the fact that some framings were used both in support of, and in opposition to, the ban. Perhaps, the combinations of framings in articles supporting or opposing the ban could have revealed a pattern? Also, I think a heat map showing which framings were used in combination would also be insightful, and may add further depth to the analysis the authors have undertaken. | We thank the reviewer for this suggestion and really liked this idea which we think enhances the presentation of the results greatly. We have added a short paragraph about the combination of frames and positions with a heat map showing these combinations. | p.8 and Figure 4 |
| Reviewer 2 |  |  |
| **Reviewer comment** | **Response** | **Change in paper** |
| This manuscript explores a relevant issue and I read it with great interest. The authors empirically analyse the use of frames concerning a ban of pesticide imports in Sri Lanka in 2021. They use a straightforward methodological approach to analyse a total of 102 media articles. The findings show a shift from the use of frames predominantly supporting the ban to more frames in opposition to the ban. |  |  |
| I have very little critique and would be happy to see this published as is. My only disappointment was that the comparison of frames used by governmental vs. private newspapers appears quite prominently in the second part of the research question and thus in the results. However, the argument for drawing this comparison is stated rather implicitly in the introduction. I would suggest to make this a bit more explicit and add a small paragraph about (specifically these) different newspaper types. | Thank you, we have added a sentence in the introduction to indicate there is research to suggest ownership impacts on the framing of news stories. | p. 4 |
| Similarly, the issue of pesticide suicide – which seems to be a primary motivation for conducting this research – is merely introduced in passing (second paragraph in the introduction). A few more words on this would help a reader not familiar with the topic. | We thank the reviewer for this comment. Whilst pesticide suicide is an important aspect for this topic, as bans were introduced in Sri Lanka to curb the exceptionally high suicide rate, this was not our main motivation for conducting the study (especially since the 2021 ban was not introduced to further reduce the suicide rate). We have added a little bit more information to the introduction to highlight its importance on the global health agenda. Given the paucity of research in this topic and region from a public health perspective our aim was to conduct an analysis characterise and examine how the problem and regulation of pesticides are frame by different actors and during a time when the ban was high on the policy and public agenda. | p. 3 |
| First, in the introduction, the statement “[…] implementing bans does not appear to compromise agricultural yield (9)” could maybe be supported by some additional references. | We thank the reviewer for this suggestion and have added some references from other countries to support this statement further. | p. 3 |
| Second, in the discussion, the authors state that “[…] it is notable that supportive frames decreased over time and opposing frames increased leading up to the revocation of the ban” (p. 15). If possible, I would be interested to read whether it can be assumed that these voices created pressure that led to the government’s decision to revoke the ban, or whether this was rather associated with an indecisiveness/ other political considerations on the part of the government. (In a very different context, this paper <https://doi.org/10.1016/j.eist.2023.100777> offers an analysis of discursive struggles in the media and the (re)creation of pesticide legitimacy.) | We thank the reviewer for this query. However, we believe that it is not possible for us to speculate on whether this pressure impacted on the government’s decision to revoke the ban. We believe this is something that should be considered in future research and would require an in-depth policy analysis using a different set of data. We have added to the final section of the strengths and limitations that further research is needed to explore how news coverage might have impacted on various stakeholders, including policy makers. We also thank the reviewer for the suggested, very interesting, article and have cited it within the discussion as we think it adds nicely to expand on that support for pesticide use, as an example, might be framed differently within different media spaces and influenced by forces beyond ownership. |  |
| Third, the section “Discussion” may also be entitled “Discussion and conclusion”, in my opinion. | The instructions to authors suggest discussion and conclusion (optional) are separate headings. We will ask the handling editor to suggest whether this section should be called ‘Discussion and conclusion’ or remain as Discussion. |  |
| Last, the manuscript entails a few typos (e.g., in this sentence on p. 4: “There is a growing body of literature exploring framings about the of prevalence…”) which the authors may wish to correct in a final round of proofreading. | We thank the reviewer for identifying this and we have undertaken a thorough proof reading before submitting the revised manuscript. |  |
