# Supplementary material for: “Ad hoc policy decisions” in the news: Media framing analysis of a pesticide import ban in Sri Lanka
Source: PLOS Glob Public Health. 2024 Jul 16;4(7):e0003497. doi: 10.1371/journal.pgph.0003497 (PMC11251609; doi:10.1371/journal.pgph.0003497)
Supplement: Attachment — Submitted filename: Responses to reviewers_R2.docx [file pgph.0003497.s003.docx]

| **Reviewer 1** | | |
| --- | --- | --- |
| **Reviewer comment** | **Response** | **Change in paper** |
| I thank the authors for taking our feedback on board, and commend them for a well written and informative paper. | We thank the reviewer again for their thoughtful read of the paper and for spotting the errors below. We really appreciate the time they’ve taken to enhance our paper. |  |
| First, it seems as if the typo in Figure 2 still remains ("the impact of the ban has on the pesticide market" is not grammatically correct, and I suggested "the impact of the ban on the pesticide market". The authors noted in their responses that they had corrected this, so perhaps they uploaded the old version when doing their resubmission. | We apologise for this, indeed the figure we uploaded still had this error which was our mistake in the upload. The correct figure is now included. | Figure 2_R2 |
| Second, on p16, "will have influence the framings" should be "will have influenced the framings" | Thank you, we have changed this. |  |
